# Vicarious facilitation of facial responses to pain: Does the others' expression need to be painful?

**DOI:** 10.1002/ejp.4709

**Published:** 2024-08-16

**Authors:** Peter J. Göller, Philipp Reicherts, Stefan Lautenbacher, Miriam Kunz

**Affiliations:** ^1^ Department of Medical, Psychology and Sociology, Medical Faculty University of Augsburg Augsburg Germany; ^2^ Bamberger LivingLab Dementia (BamLiD) University of Bamberg Bamberg Germany

## Abstract

**Introduction:**

Prior exposure to others' facial expressions of pain can lead to a facilitation of pain responses, including its corresponding response channel, namely facial responses to pain. It has been questioned, however, whether this vicarious pain facilitation occurs only when observing others' pain or whether the observation of other negative expressions can trigger similar facilitation of facial responses to pain. The study aimed to test this, by comparing the impact of viewing others' facial expressions of pain versus another negative expression (sadness) and two control expressions (neutral, happiness) on facial responses to pain.

**Method:**

Participants (*N* = 56; 31 females), watched short video clips of computer‐generated facial expressions (pain, sadness, neutral & happiness) before they received painful and non‐painful heat stimuli. Facial responses were analysed using the Facial Action Coding System. In addition, subjective and autonomic responses were assessed.

**Results:**

The prior exposure to others' expressions of pain and sadness versus neutral did not lead to significantly increased facial responses to pain. Likewise, subjective and autonomic pain responses were not facilitated. However, viewing others' expressions of happiness, consistently reduced facial as well as subjective and autonomic responses to pain compared to others' negative or neutral expressions. This dampening effect was not observed for non‐painful heat.

**Discussion:**

Facial and other pain responses were most strongly affected by prior exposure to others' facial expressions of happiness, which led to a pain‐dampening effect. In contrast, the evidence for vicarious facilitation of pain was rather weak in the present study, with no evidence of pain‐specificity.

**Significance Statement:**

Facial responses to pain – along with subjective and autonomic responses – are reduced when observing others' expressions of happiness, demonstrating pain modulation by positive affective social signals, which may also transfer to clinical contexts.

## 1 INTRODUCTION

Vicarious pain facilitation describes the phenomena that observing pain expression in others increases pain in the observer. It has been observed across various types of pain responses, including subjective, autonomic (Reicherts et al., 2013), motor (Mailhot et al., 2012; Vachon‐Presseau et al., 2011), and neural responses (Khatibi et al., 2014, 2023; Xiang et al., 2018). Vicarious pain facilitation might be a special case of motivational priming (Lang, 1995). The motivational priming theory postulates, that the display of a negative prime (e.g. others' pain expression) activates the aversive system, which leads to elevated processing of congruent stimuli (e.g. increased pain responses) (Kenntner‐Mabiala et al., 2008; Lang, 1995; Rhudy et al., 2008). Besides activation of the aversive system, pain signals might specifically pre‐activate corresponding (e.g. neural, motor) responses (Botvinick et al., 2005).

When investigating vicarious pain facilitation, photos/videos of facial expressions of pain have frequently been used as stimuli (e.g. Khatibi et al., 2023). We recently showed that these facial expression stimuli also lead to a facilitation in the corresponding response channel of the observer, namely in facial responses. Using videos of avatars displaying facial expressions of pain, we found that observing these expressions led to an increase in subsequent facial responses to pain (Göller et al., 2024). Moreover, the facilitation of facial responses was not only tied to pain versus neutral expressions but also to specific facial motor features (congruency between facial features of the prime and the corresponding pain‐induced facial muscle movements), suggesting that motor priming is also involved. Motor priming of facial responses may be closely linked to facial mimicry (Arnold & Winkielman, 2020); with both phenomena falling under the embodiment framework, according to which the perception of emotions draws upon the internal simulation of motor and somatosensory experiences (Benuzzi et al., 2018; Niedenthal, 2007).

Despite the strong evidence of pain facilitation following the presentation of facial expressions of pain, it has been questioned how specific to pain this facilitation is. Indeed, several studies found pain facilitation following exposure to negative‐affective, not pain‐related facial expressions (Bayet et al., 2014; Khatibi et al., 2023; Matamala‐Gomez et al., 2022). With regard to *facial responses* to pain, the question of whether the face stimuli necessarily need to display pain in order to elicit vicarious pain facilitation is still pending. This was the aim of the present study. Hypothetically, two outcomes might occur: (i) Comparable to other pain responses, facial responses may also be facilitated by prior exposure to negative‐affective, not pain‐related stimuli, or (ii) given that both the pain cue (others' facial expression) and the response (facial response to pain) target the same channel, the facilitation of facial responses might be more pain‐specific. To test this, videos of facial expressions of sadness and anger besides expressions of pain were included in the present study along with two control expressions (neutral, happy). Moreover, all facial expressions (positive and negative) shared at least one facial movement with the expression of pain to test for potential motor priming.

## 2 METHOD

### 2.1 Participants

Fifty‐six participants (31 female; mean age: 22.2 years) were recruited via e‐mail at the University of Augsburg. The sample included students of the University of Augsburg who received either course credit or monetary compensation (25€) for participating. All participants provided written informed consent. The study protocol was approved by the ethics committee of the University of Bamberg (#2020‐11/34).

### 2.2 Procedure

The experiment consisted of three parts. In the first part, the thermal pain threshold was determined. In the second part, the participants saw videos of avatars showing facial expressions of pain, anger, sadness, happiness, or a neutral expression prior to receiving painful and non‐painful thermal stimuli. In order to distract participants from the aim of the study, they had to count the number of female faces. The facial responses to the thermal stimuli were recorded via video. Additionally, pain intensity and pain unpleasantness ratings as well as skin conductance responses were assessed. The pain induction protocol used in the first and second parts follows our previous study where we could successfully show vicarious facilitation of facial responses to pain (Göller et al., 2024). In the third part, participants evaluated the emotional impact of the used facial expression stimuli by providing valence and arousal ratings for each facial expression. The experiment lasted for approximately 70 min.

### 2.3 Pain induction

Thermal stimuli were applied using the TSA II (Peltier‐based contact stimulation device (TSA‐2001, Medoc, Israel)) with a 30 × 30 mm contact thermode that was attached to the outside of the left lower leg with a gauze bandage.

To ensure that temperature intensities were perceived as painful but not too painful in all participants (to prevent floor as well as ceiling effects), temperature intensities were tailored to the individual pain threshold. Thus, heat pain thresholds were determined first, using the method of adjustment. Participants were asked to adjust a temperature starting from 38°C, heating and cooling the thermode by button presses, until they obtained a level that was perceived as barely painful. A constant press of the buttons produced a heating or cooling rate of 0.5°C/s. Following a familiarization trial, there were four trials and the average of these four trials was used to constitute the threshold estimate.

Following the assessment of pain thresholds, phasic heat stimuli (trapezoid form, 5 s [plateau]; rate of change: 4°C/s; baseline temperature: 38°C) were applied using two different stimulus intensities, namely painful (+3°C above each participant's individual pain threshold) and non‐painful (−1°C below each participant's individual pain threshold). Given that the average pain‐threshold in our sample was 45.4°C (SD 0.6°C), mean temperatures for non‐painful and painful heat stimulation were 44.4°C and 48.4°C, respectively.

There were 60 quasi‐randomized heat stimuli (40 painful, 20 non‐painful) split up into three blocks (21 trials, 21 trials, and 18 trials). For each block, the position of the thermode was slightly changed (by moving it upwards or downwards in a randomized order) in order to prevent sensitization.

### 2.4 Facial expression stimuli (others' expression)

**FIGURE 1:**
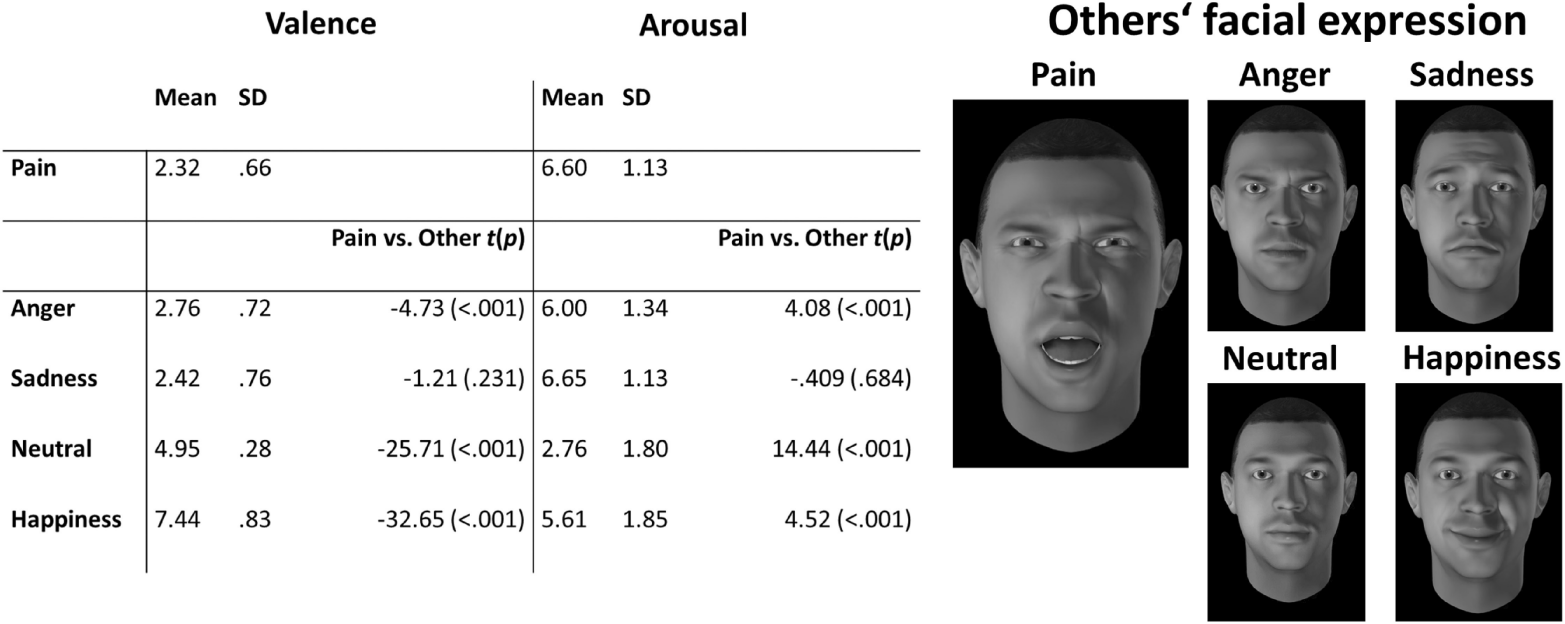
Mean score and SD for Valence and Arousal ratings (Pain rating (SAM; 1–9)) for the facial expression stimuli. T‐values of paired *t*‐tests comparing the facial expression of pain stimuli to the other facial expressions with corresponding *p*‐value. Additionally, the stimulus material is presented herein (the male avatar) displaying the five facial expressions (pain, anger, sadness, happiness, and neutral).

The faces of the avatars were modelled with the software FaceGen Modeller Core 3.5 (Version of 2019). The used avatars had different hairstyles and different skin colour. The videos of different dynamic facial expressions were created with the additional software FACSGen3 (Version of 2019), for which Krumhuber and Tamarit (Krumhuber et al., 2012) and Roesch et al. (Roesch et al., 2011) have demonstrated that it produces emotionally valid and reliable facial expressions. The facial expressions being created in FACSGen3 are based on the Facial Action Coding System (FACS; Ekman & Friesen, 1978) which distinguishes 44 different “Action Units” (AUs). For this study, we created a prototypical facial expression of pain based on the review by Kunz et al. (2019), which included AU4; AU6_7; AU9_10; AU25_26_27(see example in Figure 1). As other negative valent facial expressions, we also created expressions of sadness and anger, given that facial expressions of pain have been shown to be blended as well as to be mistaken for anger as well as for sadness and thus, seem to show high proximity (Hale & Hadjistavropoulos, 1997; Kappesser & de Williams, 2002; LeResche & Dworkin, 1988). The facial expression of sadness was created by combining AU1; AU4; AU15 and the facial expression of anger was created by combining AU4; AU5; AU6_7; AU24 (based on Ekman & Friesen, 1978). Moreover, an expression of happiness (combining AU6_7 and AU12) and a neutral expression (only included an eye‐blink, AU 45) were created as control conditions. We tested these customized avatar expressions in previous studies and showed that observers could correctly decode the intended affective states (Göller et al., 2023; Meister et al., 2021). Each of the five dynamic expressions (pain, anger, sadness, happy, neutral) was displayed by one male and one female avatar using identical facial activity patterns.

Each video had a duration of 5000 ms (neutral baseline 1500 ms, unfolding of the expression for 1000 ms, full expression for 500 ms, decline again for another 1000 ms, and another neutral baseline for 1000 ms). Each trial started with the appearance of a fixation cross (white cross on a black background) for 10 S. The videos started 1500 ms before the start of the heat stimulation, so that the unfolding of the expression started in parallel to the start of the ramp‐up of the heat stimulation (see Figure S1 showing the stimulation protocol). We timed it in such a way that the end of the dynamic facial expressions directly preceded the 5 s plateau phase of the painful heat stimuli, given that the time between prime and painful stimulus is critical and should be very short (<500 ms; (Richter et al., 2014)). The videos were presented using the software “Presentation” (Neurobs, Version 21.1). Altogether, 10 videos were created (5 expressions x 2 avatars (male/female)) and each video was presented six times in a quasi‐randomized order, balanced across painful (*N* = 40) non‐painful (*N* = 20) heat stimuli.

As stated above, we masked the primary aim of the study by instructing participants to count the number of female faces in each block. The compliance rate was very high, with most participants reporting the correct numbers (block 1: 95%, block 2: 91%, and block 3: 90% of the participants).

### 2.5 Pain ratings

After each heat stimulus, participants were asked to rate the intensity & unpleasantness of pain via visual analogue scales (VAS). The pain intensity scale reached from “no pain” to “extremely strong pain” and the pain unpleasantness scale reached from “no pain” to “extremely strong unpleasantness”. The two scales (ranging from 0 to 100 points) appeared together on the computer screen and participants moved a slider to indicate their ratings. To familiarize subjects with the rating procedure, one practice trial was conducted.

### 2.6 Facial responses to pain

Participants' faces were videotaped throughout the heat stimulation. The camera was located approximately 2 m in front of the participant to allow for a frontal view. To enable the offline segmentation, a sound trigger (a bing‐sound) was used in the video recording that marked the start of the thermal stimulation, as well as the start and end of the plateau phase. The sound trigger was not audible to the participants. To ensure that the face would always be upright and in a frontal view during stimulation, participants were asked to avoid movements and to look at the computer screen. Participants were also instructed to avoid talking during the experiment.

Facial responses were coded from the video recordings using the Facial Action Coding System (FACS) (Ekman & Friesen, 1978). FACS is grounded on an anatomical analysis of facial movements and distinguishes 44 different “Action Units” (AUs) produced by single muscles or combinations of muscles. A certified FACS coder (qualified by passing an examination given by the developers of the system) who was blind to the experimental conditions identified the frequency and the intensity (five‐point scale) of the different AUs. In order to determine interrater reliability, 5% of the video segments were coded by a second‐certified FACS coder also blinded to the experimental conditions. Interrater reliability was calculated using the Ekman–Friesen formula (Ekman & Friesen, 1978). Interrater reliability was *r* = 0.76 which compares favourably with other research in the FACS literature (e.g. (Karmann et al., 2018; Priebe et al., 2015)). A software designed for the analysis of observational data (Observer Video‐Pro; Noldus Information Technology, Netherlands) was used to segment the videos and to enter the FACS codes into a time‐related database.

Time segments of 7 s beginning just after the stimulus had reached the target temperature were selected for scoring. In total, 60 segments of heat stimulation (40 painful segments and 20 non‐painful) were analysed for each participant. Pain‐specific AUs were selected based on a review article on facial expressions of pain (Kunz et al., 2019) and included the following Action Units: AU4, AU6_7, AU9_10, and AU25_26_27. For later analyses, the two FACS parameters, namely mean intensity and sum frequency values of each pain indicative AU were combined by multiplication (product terms).

### 2.7 Skin conductance response

We additionally assessed the skin conductance level (SCL, a measure of sympathetic nervous system activation) as an autonomic pain response in the study. Time segments of 8.5 s, beginning just after the stimulus had reached the target temperature, were selected for scoring. This time window is slightly longer compared to the analysis window for facial responses given that the skin conductance response occurs with a slight delay. For skin conductance recording, two 22/10 mm Ag/ AgCl surface electrodes (electrode gel: 0.5% NaCl) were attached to the thenar and hypothenar eminence of the participant's nondominant hand. The signal was sampled with 250 Hz, with a constant application of 0.5 V, using a V‐Amp amplifier (Brain Products Inc, Munich, Germany) and the recording software Brain Vision Recorder (Brain Products Inc) and was offline down‐sampled to 20 Hz. Data was averaged across all trials per condition (five types of others' facial expression stimuli and the two heat intensities). For baseline correction, the 1‐s intervals preceding the onset of the facial expression stimuli were chosen. After this, the baseline‐corrected mean SCL to each thermal stimulus was calculated within a time window of 8.5 s (starting with stimulus reaching target intensity). One participant was excluded from the SCL – data analysis because of fragmented data.

### 2.8 Evaluation of the facial expression stimuli: Valence and arousal ratings

At the end of the study, participants were asked to provide valence and arousal ratings of all *N* = 10 facial expression stimuli (neutral, happiness, anger, sadness, pain; for male and female avatars). Valence and arousal were assessed using Self‐Assessment Manikin (SAM (Bradley & Lang, 1994)) that appeared in the middle of the computer screen (valence on the upper half, arousal on the lower half). Ratings were performed by mouse click on the manikins or spaces in‐between, resulting in nine categories (i.e., “maximum positive” = 1 to “maximum negative” = 9 for valence and “maximum” = 9 to “no” = 1 for arousal). The participants had unlimited time to provide their ratings. Only after participants provided the two ratings, they were able to click on the button “continue” and the next facial expression of the avatars unfolded and had to be rated. Ratings were performed to make sure that (i) the negative expressions (anger, sadness) were perceived as similar to pain expressions in terms of valence and arousal and (ii) that the control expressions (happiness and neutral) were rated as more positive and less arousing than the pain expressions.

### 2.9 Statistical analysis

#### 2.9.1 Manipulation check

In order to compare valence and arousal ratings of the facial expression stimuli (pain, anger, sadness, happiness, and neutral), we calculated repeated measure ANOVAs including the within‐subject factor “avatars' facial expression” (pain, anger, sadness, happiness, neutral), separately for valence and arousal ratings. In case of a significant effect, post‐hoc comparisons were performed.

### 2.10 Effect of the facial expression stimuli (others' expression) on pain responses

#### 2.10.1 Pain ratings

To investigate whether the prior exposure to others' expression affected the VAS ratings for non‐painful and painful heat intensities, pain intensity and unpleasantness ratings were analysed using a repeated measure MANOVA including the within‐subject factors “others' facial expression” and “heat intensity”.

#### 2.10.2 Facial responses

To investigate the effect of others' expression on facial responses to non‐painful and painful heat intensities, we entered the pain‐specific Action Units (AU4, AU6_7, AU9_10, AU25_26_27) into a repeated measure MANOVA including the within‐subject factors “others' facial expression” and “heat intensity”.

#### 2.10.3 SCL

To investigate the effect of others' expression on skin conductance level changes to non‐painful and painful heat intensities, a repeated measures ANOVA including the within‐subject factors “others' facial expression” and “heat intensity” was performed.

For all described repeated measure ANOVAs, a Greenhouse–Geisser correction was used in case of violation of sphericity assumption. In case of significant effects, post‐hoc analyses were conducted for single comparisons.

All analyses were conducted with SPSS 28, and the alpha level was 0.05 (α) throughout.

## 3 RESULTS

### 3.1 Manipulation check

The rm‐ANOVAs showed a significant main effect for others' facial expression on valence (*F*(2.5, 132.5) = 627.99 *p* < 0.001, η^2^ = 0.921) and arousal ratings (*F*(2.6, 140.4) = 101.99 *p* < 0.001, η^2^ = 0.654). For single comparisons, the post‐hoc *t*‐test were computed to compare the pain expression stimuli to the other expressions (see Figure 1). As intended, the positive and neutral facial expression stimuli elicited more positive valence and less arousal compared to the pain expression stimuli. With regard to the negative stimuli, we observed comparable valence and arousal ratings for pain and sadness expressions. However, valence and arousal ratings for the anger stimuli differed significantly from the stimuli showing “facial expressions of pain” (see Figure 1). This observation contradicted the objectives of our study, as the perception of others' negative facial expression stimuli should not exhibit disparities in valence and arousal compared to the pain expression stimuli. Consequently, we opted to exclude the facial expression of anger from further analyses.

### 3.2 Effect of the facial expression stimuli (others' expression) on pain responses

#### 3.2.1 Pain ratings

**FIGURE 2:**
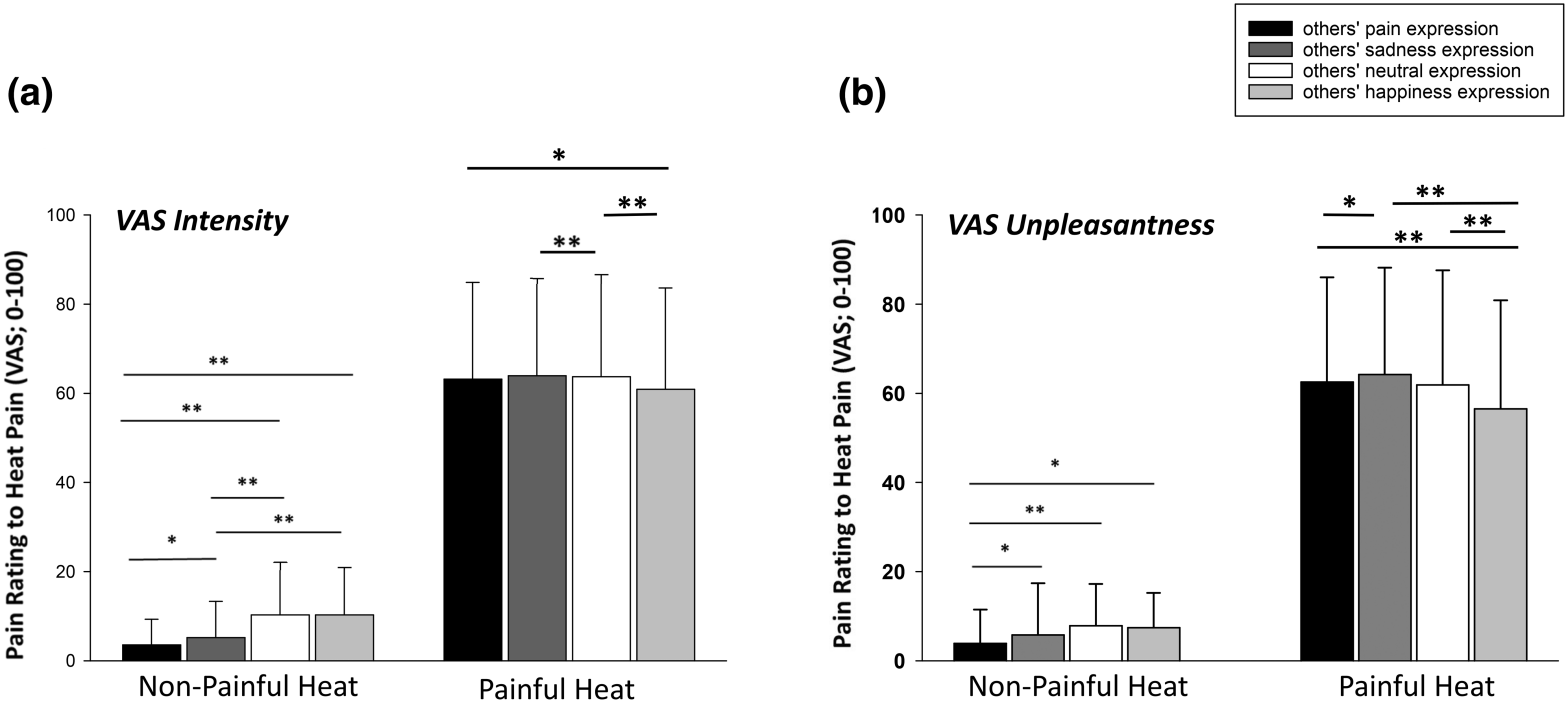
Effect of “facial expression stimuli” on pain intensity (a) and unpleasantness (b) ratings (mean, SD). Ratings are presented separately for non‐painful and painful heat intensities as well as separately for the different others' facial expression stimuli. *p < 0.05; **p < 0.001; SD, standard deviation.

The rm‐MANOVA showed a significant main effect for “heat intensity” on pain ratings (*F*(2, 52) = 224.20, *p* < 0.001, η^2^ = 0.896), with pain ratings significantly increasing from non‐painful to painful heat stimulation (see Figure 2a,b). Univariate outcomes showed that these significant effects were found for both types of pain ratings, namely for pain intensity as well as unpleasantness ratings. In detail, both ratings significantly increased across non‐painful to painful heat intensities (VAS intensity: *F*(1, 53.0) = 451.40, *p* < 0.001, η^2^ = 0.895; VAS unpleasantness: *F*(1, 53.0) = 351.99, *p* < 0.001, η^2^ = 0.869).

Moreover, “others' facial expression” had a significant effect on pain ratings (*F*(6, 316) = 9.47; *p* < 0.001, η^2^ = 0.154). As univariate outcomes showed, this significant effect could be found for both VAS intensity (*F*(2.3, 123.6) = 12.97, *p* < 0.001, η^2^ = 0.197) and VAS unpleasantness ratings (*F*(1.7, 123.6) = 4.92, *p* = 0.014, η^2^ = 0.085). Furthermore, a significant interaction between “others' facial expression” and “heat intensity” was also found (*F*(6, 316) = 12.18, *p* < 0.001, η^2^ = 0.188); again for both VAS intensity (*F*(2.5, 130.8) = 20.30, *p* < 0.001, η^2^ = 0.277) and VAS unpleasantness ratings (*F*(2.5, 134.2) = 22.5, *p* < 0.001, η^2^ = 0.296). Post‐hoc *t*‐tests were computed for simple comparisons separately for non‐painful and painful heat intensities and the significant results are displayed in Figure 2a (VAS intensity) and 2b (VAS unpleasantness). With regard to ratings of non‐painful heat intensities (left side of Figure 2a,b), we found that the prior exposure to negative facial expressions (especially pain) surprisingly led to lower pain ratings. With regard to ratings of painful heat intensities, we found that especially positive facial expressions (happiness) led to a significant decrease in pain intensity and unpleasantness ratings compared to prior exposure to a negative or neutral facial expressions.

#### 3.2.2 Facial responses

**FIGURE 3:**
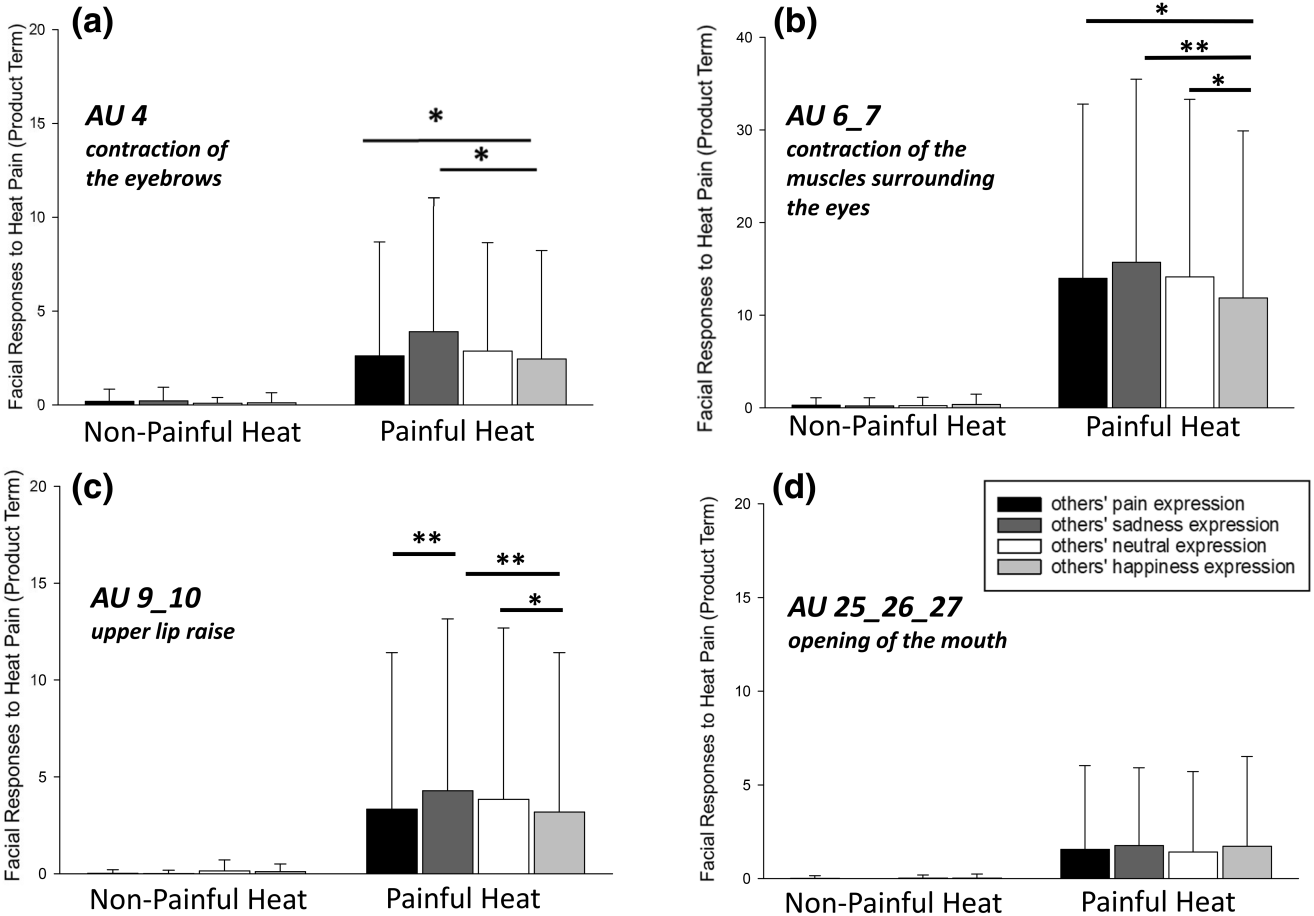
Effect of “others' facial expression” on facial responses (pain‐specific AUs mean, SD) to heat stimulation. Facial responses are presented separately for non‐painful and painful heat intensities as well as separately for trials with prior exposure to the different others' facial expression stimuli. *p < 0.05; **p < 0.001; AUs, Action Units; SD, standard deviation.

The rm‐MANOVA showed a significant main effect for “heat intensity” on facial responses (*F*(4, 50) = 224.20, *p* < 0.001, η^2^ = 0.380), with facial responses significantly increasing from non‐painful to painful heat stimulation (see Figure 3a–d). Univariate outcomes showed that all pain‐specific AUs significantly increased across non‐painful to painful heat intensities (AU4: *F*(1, 53.0) = 12.85, *p* < 0.001, η^2^ = 0.195; AU6_7: *F*(1, 53.0) = 30.16, *p* < 0.001, η^2^ = 0.363; AU9_10: *F*(1, 53.0) = 10.10, *p* = 0.002, η^2^ = 0.160; AU25_26_27: *F*(1, 53.0) = 7.71, *p* = 0.008, η^2^ = 0.127).

The rm‐MANOVA also showed a significant main effect for the factor “others' facial expression” (pain, sadness, happiness, neutral) on facial responses (*F*(12, 413.0) = 2.62, *p* = 0.002, η^2^ = 0.063). Univariate outcomes showed, that this effect was significant for three out of the four pain‐specific AUs, namely AU 4: *F*(2.2, 116.8) = 3.67, *p* = 0.025, η^2^ = 0.065, AU 6_7: (*F*(2.6, 140.0) = 4.47, *p* = 0.007, η^2^ = 0.078 as well as AU9_10: *F*(2.5, 131.6) = 5.41, *p* = 0.003, η^2^ = 0.093). Only AU 25_26_27 (opening of the mouth) was not affected by prior exposure to “others' facial expression” (*F*(2.3122.9) = 0.65, *p* = 0.584, η^2^ = 0.012).

Furthermore, a significant interaction between “others' facial expression” and “heat intensity” was also found (*F*(12,413.0) = 280, *p* < 0.001, η^2^ = 0.068); again for three out of the four pain‐specific AUs; namely AU 4: *F*(2.2, 114.5) = 3.34, *p* = 0.036, η^2^ = 0.059, AU 6_7: *F*(2.7, 144.2) = 5.77, *p* = 0.001, η^2^ = 0.098 as well as AU9_10: *F*(2.6, 138.6) = 7.13, *p* < 0.001, η^2^ = 0.119.

Post‐hoc *t*‐tests were computed for simple comparisons separately for non‐painful and painful heat intensities and the significant results are displayed in Figure 3a–d. With regard to facial responses to non‐painful heat intensities (left side of Figure 3a–d), we found that the prior exposure to “others” facial expression had no effect on facial responses. In contrast, facial responses to painful heat (right side of Figure 3a–d) were significantly affected by prior exposure to others' facial expressions. Especially viewing a happiness expression, resulted in significantly decreased pain‐specific facial responses to pain (AU4, AU 6_7 and AU 9_10) compared to prior exposure to pain, sadness, and neutral stimuli.

#### 3.2.3 Skin conductance level (SCL)

**FIGURE 4:**
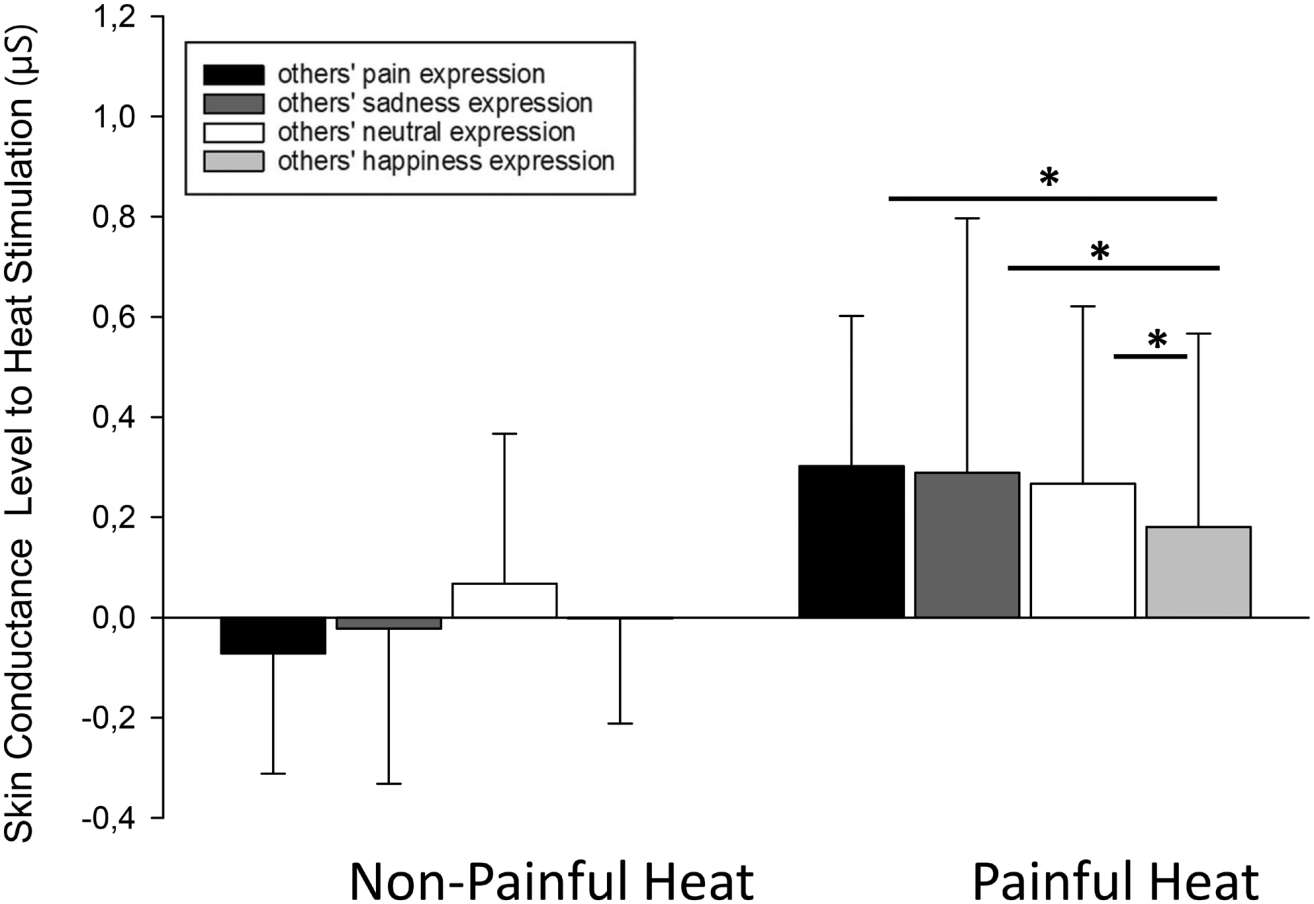
Effect of “others' facial expression” on skin conductance response (baseline‐corrected, mean, SD) (Ln(SCL + 1) [μS]) to heat stimulation. Mean skin conductance responses are presented separately for non‐painful and painful heat intensities as well as separately for trials with prior exposure to the different facial expression stimuli. *p < 0.05; SD, standard deviation.

The rm‐ANOVA showed a significant main effect for “heat intensity” on SCL (*F*(1, 53.0) = 43.48, *p* < 0.001, η^2^ = 0.451), with SCL significantly increasing from non‐painful to painful heat stimulation (see Figure 4). Although we found no significant main effect for “others' facial expression” on SCL (*F*(2.6, 138.8) = 1.96, *p* = 0.131, η^2^ = 0.036); there was a significant interaction between “heat intensity” and “others' facial expression” (*F*(2.0, 104.8) = 3.40, *p* = 0.038, η^2^ = 0.060). Post‐hoc *t*‐tests were computed for simple comparisons separately for non‐painful and painful heat intensities and the significant results are displayed in Figure 4. With regard to non‐painful heat intensities (left side of Figure 4), we found that the prior exposure to “others” facial expression had no effect on SCL. In contrast, SCL response to painful heat intensities was significantly affected by prior exposure to others' facial expressions. Viewing a happiness expression elicited lower pain‐related SCL changes compared to negative (sadness and pain) or neutral facial expression stimuli (Figure 4).

#### 3.2.4 Summary

In contrast to our expectation, the prior exposure to others' negative facial expressions (pain and sadness) did not lead to a facilitation in pain responses compared to prior exposure to neutral expressions. Only the prior exposure to others' facial expressions of happiness had a stable dampening effect on all responses to painful heat, with decreased pain ratings, decreased pain‐specific facial responses, and decreased skin conductance levels following heat pain stimulation. Furthermore, this effect became evident only for painful heat. During non‐painful trials, prior exposure to happy expressions did not lead to a reduction of facial, subjective, or autonomic responses (in case of pain ratings, we even found elevated pain ratings after viewing happy expressions).

## 4 DISCUSSION

In the context of vicarious facilitation of facial responses to pain, our aim was to test whether the others' expression needs to be painful or whether similar facilitation might also be elicited by other negative expressions. In contrast to our expectations, neither the prior exposure to others' expressions of pain nor to others' expressions of sadness led to a clear facilitation of facial responses to pain compared to others' neutral expressions. Instead, only the prior exposure to others' expressions of happiness led to a pain‐dampening effect, with reduced facial responses to pain compared to prior exposure to negative and neutral facial expressions. Similarly, pain‐dampening effects were observed for subjective and skin conductance responses to painful heat stimulation; whereas happy expressions had no dampening effects on responses to non‐painful heat. We will discuss these findings in detail below.

### 4.1 Vicarious facilitation of pain responses

In the present study, the prior exposure to others' expressions of pain versus neutral expressions did not lead to the expected increase in facial responses; neither to non‐painful nor painful heat stimulation. Similarly, subjective and autonomic responses also showed no indication of vicarious pain facilitation. Effect sizes computed suggest negligible effects (η^2^ < 0.01) for vicarious pain facilitation in the present study. This is in contrast to a previous study of our group where we found clear evidence (large effect sizes η^2^ > 0.14) of vicarious facilitation of facial and subjective responses to pain (Göller et al., 2024). A reason for the differing findings might be the characteristics of the facial expression stimuli. In the previous study, only two types of facial expressions, namely pain and neutral, were used, whereas the range of affective states was broadened in the present study and two other emotional categories were employed. Specifically, besides pain and neutral expressions, we additionally included others' facial expressions of sadness and anger as well as a positive facial expression, namely happiness. It is possible that the elevated number of negative stimuli (pain, sadness, anger) affected how the neutral facial expression was perceived. Indeed, it has been shown that others' neutral facial expressions are perceived as negative when preceding negative expressions served as primes (Chiesa et al., 2017; Höschel & Irle, 2001; Jellema et al., 2011; Lu et al., 2011). Since these negative primes occurred more frequently in the present study compared to our previous study, it is possible that the neutral facial expression was also perceived as more negative during the pain testing; although the later assessed valence ratings did not show this. Besides the larger frequency of negative primes, the overall number of distinct affective states might also have played a role. Prior studies showing vicarious facilitation of pain have typically compared two (Ibáñez et al., 2011; Van Middendorp et al., 2010) or three types of others' expressions (Bayet et al., 2014; Khatibi et al., 2023). It is plausible that using five types of facial expressions in the present study design might have overloaded the participants; in other words, vicarious facilitation of pain might be more evident when using a limited number of facial expressions as primes. It is also possible that using avatars instead of real human facial expressions might have contributed to the lack of vicarious facilitation of pain.

In sum, we included five different types of facial expressions as primes in the present study to be able to investigate the pain specificity of vicarious facilitation of facial responses to pain. However, this increase in the number and variability of others' expressions might have unfortunately dampened the well‐known pain facilitation effect that we aimed to investigate. Hence, it would have been preferable to conduct separate studies, each study only including two or three maximal types of facial expression stimuli. A limited set of two facial expressions preceding pain stimulation also has the advantage that this design seems to align more with real‐life situations than the more complex priming paradigm used in the present study.

### 4.2 Positive priming effect on pain responses

We found a clear pain‐dampening effect when participants viewed others' facial expression of happiness prior to painful heat stimulation. This dampening effect was apparent in facial responses as well as in subjective and autonomic responses to pain. Similar pain‐dampening effects have been observed in previous studies using facial expression of happiness as primes preceding a painful stimulation. More precisely this has been observed for neural (Kenntner‐Mabiala & Pauli, 2005; Kornelsen et al., 2019; Orenius et al., 2017) subjective (Matamala‐Gomez et al., 2022; Mini et al., 1995; Wieser et al., 2014), and autonomic (Reicherts et al., 2013; Roy et al., 2011) responses to pain and now, also for facial responses to pain. According to Lang's (Lang, 1995) motivational priming theory, a positive or pleasant prime is capable of activating the motivational approach system and by that to inhibit – in terms of valence – incongruent response, which is the case when a smiling face or a pleasant picture is paired with an aversive pain stimulation (Hale & Hadjistavropoulos, 1997; Kenntner‐Mabiala & Pauli, 2005; Meagher et al., 2001; Meng et al., 2012). Interestingly and in line with our findings, a recent meta‐analysis on the effect of emotion induction on pain showed strong pain‐dampening effects for positive emotions whereas findings for negative emotion induction were less clear, with only a few studies showing pain‐facilitation effects whereas others failed to do so (Mikkelsen et al., 2024). Besides motivational priming, our findings can also be explained using the embodiment framework given that we used facial expressions as stimuli. According to the embodied perception perspective (e.g. Henrich et al., 2007), the perception of happy expressions should trigger the activation of the observer's own viscero‐motor (e.g. facial mimicry) or somatosensory representation of happiness and hereby dampen the following pain experience.

In our study, this effect may be pronounced due to the facial expression of happiness assuming the role of an oddball. This is attributed to its infrequent occurrence as a positively valenced facial expression compared to the more common negative valenced facial expressions (12 vs. 36). Consequently, the others' expression of happiness might be more attention‐grabbing, thereby contributing to the observed pain‐dampening effect.

### 4.3 Motor priming of facial responses to pain

Indications of motor priming observed in our previous study (Göller et al., 2024) were not replicated in the current study. The facial expression stimuli used in the present study shared at least one Action Unit with the known subset of facial responses to pain (Kunz et al., 2019) (e.g. AU 6_7 belongs to the pain‐specific AUs and is also part of the happiness expression). If motor priming plays a role, we expected that facial responses to pain might be altered depending on which AU was present in the preceding prime (e.g. AU 4 should be especially facilitated following the exposure to facial expressions of sadness and pain). In contrast to our expectation, we did not find that single AUs were differentially affected by the different facial expression stimuli. Therefore, motor priming effects could not be found in the present study. It is possible that we could not replicate the motor priming effect as found in the previous study (Göller et al., 2024) due to similar reasons as discussed above (number and variety of facial expression stimuli). As a limitation, we only assessed visible facial muscle movements with the FACS and thus, cannot exclude that more subtle motor priming of facial responses might have occurred that would have been captured using electromyography (EMG) to record facial responses to pain.

### 4.4 Clinical implications

Our findings might be especially relevant for pain settings where a generally negative valence prevails. In these settings, positive stimuli could be utilized to potentially reduce pain. Examples include post‐operative settings, where pain and other negative valenced states (e.g. nausea, feeling disoriented, anxiety) are prevalent. In such settings, positive images might have a pain‐dampening effect. Similarly, in facilities treating chronic pain patients, where negative valence might predominate (e.g. due to the long‐standing suffering of the patients), the display of positive images might contribute to pain‐dampening effects.

## 5 CONCLUSION

We could show that a prior exposure to positive others' expression lead to decreased pain responses, manifesting across all investigated response channels (facial, subjective, autonomic) of pain. In contrast to our expectation, we found no clear evidence of vicarious facilitation of pain, possibly due to the large number of different primes used in the present study. Thus, a simpler design might be necessary to answer the question of how pain‐specific vicarious facilitation of facial responses to pain is.

## AUTHOR CONTRIBUTIONS

This study was designed by M.K., S.L., and P.R. The experiment was performed by P.G. The data was analysed by P.G. and the results were critically examined by all authors. P.G. and M.K. had a primary role in preparing the manuscript, which was edited by S.L. and P.R. All authors have approved the final version of the manuscript and agree to be accountable for all aspects of the work.

## Supporting information


Figure S1.
