# Supplementary material for: Vicarious facilitation of facial responses to pain: Does the others' expression need to be painful?
Source: Eur J Pain. 2024 Aug 16;29(1):e4709. doi: 10.1002/ejp.4709 (PMC11609883; doi:10.1002/ejp.4709)
Supplement: Supplementary file 1 — Figure S1. [file EJP-29-0-s001.docx]

**Supplementary:**


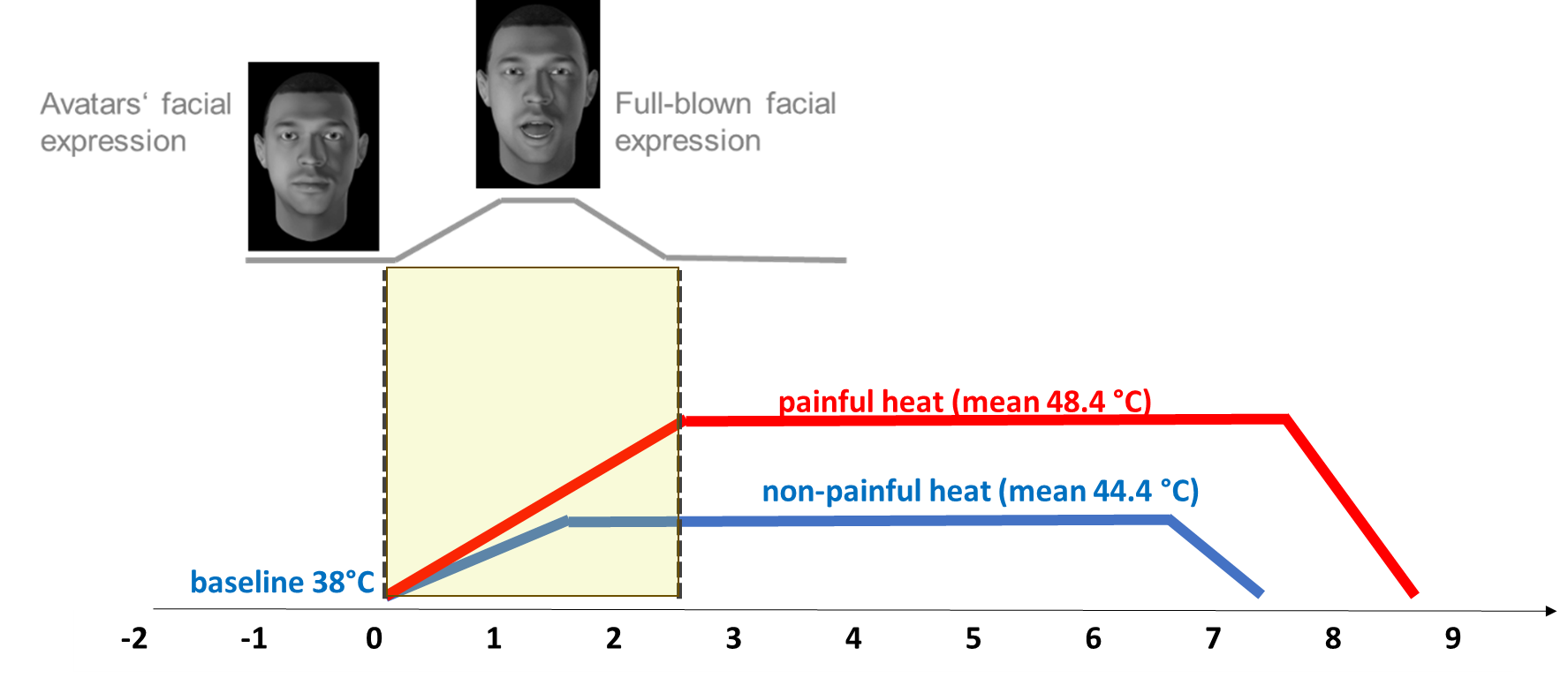


***Figure S1.*** *Stimulation protocol to display the temporal sequence of the facial expression primes and the thermal heat stimulation (separately for non-painful and painful heat intensities).*
